# A feature-centric predictive and sensitivity analysis framework for bio-oil yield assessment in biomass pyrolysis

**DOI:** 10.1038/s41598-026-65385-9

**Published:** 2026-08-09

**Authors:** Shahad Almansour, Lulwah M. Alkwai, Kusum Yadav, Debashis Dutta

**Affiliations:** 1 Applied College, University of Ha’il, Ha’il, Kingdom of Saudi Arabia; 2 College of Computer Science and Engineering, University of Ha’il, Ha’il, Kingdom of Saudi Arabia; 3 Sharda School of Engineering and Sciences, Sharda University, Greater Noida, UP, India; 4 Department of computers Techniques engineering, College of technical engineering, The Islamic University, Najaf, Iraq

**Keywords:** Bio-oil yield prediction, Feature selection, Sensitivity analysis, Biomass pyrolysis, Model interpretability, Energy science and technology, Engineering, Environmental sciences, Mathematics and computing

## Abstract

Accurately predicting bio-oil yield from biomass pyrolysis is a real challenge due to nonlinear interactions between feedstock physicochemical properties and operating conditions. In addition, high feature dimensionality, uncertainties in experimental measurements, and multicollinearity make prediction accuracy and interpretability even more difficult, which explains the demand for a feature-driven and explainable modeling framework. We developed an advanced regression feature-based machine learning framework trained on a very comprehensive dataset of both biomass characteristics and pyrolysis conditions. Correlation-based feature screening was performed to identify informative variables and examine potential redundancy among input parameters before predictive modeling. The model’s performance in the training, validation, and testing phases was assessed using a variety of statistical metrics. To keep the model understandable, global sensitivity analysis and feature attribution methods were used to determine the relative impacts of each input and their combined effects on yield variation. The suggested system design attained very accurate predictions, demonstrating a test-stage coefficient of determination over 0.91 and consistently low prediction errors. Sensitivity analysis identified working temperature and volatile content as the key substrates for yield, followed by fixed carbon and ash content, while elemental hydrogen and oxygen showed condition-dependent behavior. The agreement in feature scoring between sensitivity methods demonstrates the reliability of the concept-based interpretation. The primary novelty of the paper is incorporating feature selection, predictive modeling, and sensitivity analysis into one feature-oriented yield prediction framework. Unlike purely accuracy-driven studies, this approach simultaneously enhances predictive performance and interpretability, providing quantitative insight into feature importance and system sensitivity.

**Supplementary Information:**

The online version contains supplementary material available at https://doi.org/10.1038/s41598-026-65385-9.

## Introduction

Yield is a key performance metric that specifies the amount of productive output a system generates relative to its input conditions. In fact, yield is one of the most widely used and versatile parameters of product quality in scientific and technological fields. It is first and foremost a complex concept that cannot be attributed to one factor only. The yield is the result of the continuous and nonlinear interplay of all the variables that affect it^1^. In fact, yield depends on the interactions of several process variables, which may be nonlinear and time-varying in nature. Besides, each variable (or feature) characteristically contributes with a different weight to the final result^2^. According to recent publications, the sensitivity of yield to changes in features and their interactions is remarkable. Input features are typically dissimilar in scale, range, and behavior over time. Moreover, the connection between input features and yield is commonly both nonlinear and nonstationary^3^. Thus, yield data usually have high dimensionality and multicollinearity and contain noise, which make an accurate yield prediction difficult^4,5^. Hence, one has to perform local decomposition and carefully examine the contributions and interactions of individual features to yield variations^6^.

Uncertainty in measured yield is a major reason yield research has remained so challenging. Measurement errors, environmental factors, and operational variations are all sources of feature variability that impact yield reliability^7,8^. Yield variations result from a variety of sources, and their effects superimpose in the feature space. For this reason, present-day research in this field largely focuses on representing yield variability as a function of feature uncertainties while maintaining a suitable degree of accuracy^9^. Interpretability of yield models along with their accuracy is what stakeholders nowadays most value in yield analysis. In fact, the first question that the majority of stakeholders ask when deciding whether to invest is: ‘Who controls the yield and how?‘. It is their basic right to know how the process variables affect yield and what each variable contributes^10,11^. Evaluating the degree of feature contribution and sensitivity allows one to discern which features are the major influencers, minor contributors, or negligible factors. This information not only supports data-driven decision-making but also opens avenues for process optimization and risk management^12^. Currently, feature studies in yield mainly consider yield as a variable, a resultant effect, or a feature-based outcome. Yield is often regarded as a feature-centric entity whose analysis, although dependent on feature relevancy and their interactions, is independent of the features themselves^13,14^.

Malashin et al. accomplished a high level of accuracy in predicting crop yields with the help of a deep neural network (DNN) combined with a genetic algorithm (GA) based hyperparameter optimization, and they also demonstrated how LIME, an explainable AI (XAI), helped them figure out which features were most influential^15^. In their review of deep learning and ensemble models, Shawon et al. revealed that DNNs, CNNs, and other machine learning approaches can beat traditional regressions because they can uncover nonlinear interactions among environmental, soil, and management factors^16^. The role of deep learning in this area is once again emphasized by Wang et al., who, after reviewing the development of DNN-based methods for crop yield prediction, single out these methods as better than traditional models in identifying complex feature interactions and capturing temporal dynamics^18^. Li et al. implemented XGBoost combined with multi-source data to forecast winter wheat output and employed SHAP to explain feature importance. Their research reveals that tree-based ensemble models not only have the potential to deliver highly accurate predictions but can also offer insights into the main features impacting yield, thus pointing to the benefit of explainable machine learning in precision agriculture^17^. Meanwhile, Najjar et al. show how multimodal models with attention mechanisms and SHAP attributions lead to better predictive accuracy and interpretability in yield forecasting^18^.

In addition to predictive modeling, feature dimensionality reduction and feature extraction techniques have become increasingly important for handling the complexity of biomass pyrolysis data. Since bio-oil composition and yield are governed by numerous interacting chemical and operational variables, extracting informative features while reducing redundant information is essential for improving model reliability and interpretability. Recent studies have demonstrated the effectiveness of combining analytical characterization techniques with machine learning approaches for pyrolysis product prediction. For example, ATR-FTIR spectroscopy combined with machine learning models has been used to rapidly characterize biomass pyrolysis oil, demonstrating that spectral information can provide valuable predictive signatures for complex bio-oil properties^19^. Similarly, integrated machine learning frameworks coupled with infrared spectroscopy have been developed to achieve interpretable pyrolysis oil characterization by identifying influential spectral features and establishing relationships between chemical information and product characteristics^20^. These studies highlight the importance of feature representation and dimensionality reduction in improving data-driven pyrolysis analysis.

**Fig. 1:**
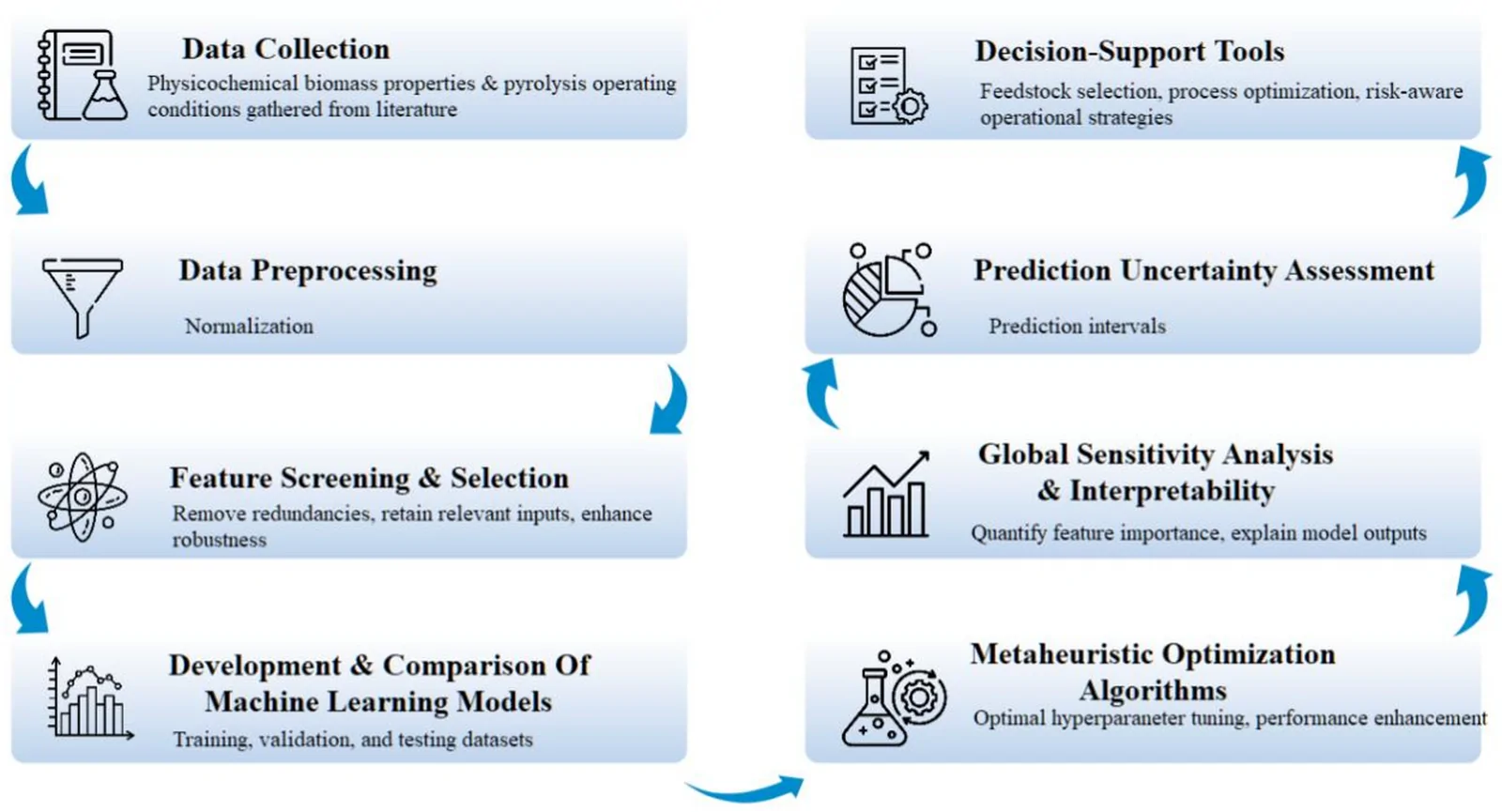
Comprehensive machine learning workflow for biomass pyrolysis modeling and decision support.

This paper presents a feature-centric integrated framework that merges precise yield prediction with systematic feature selection and sensitivity analysis, thus achieving both high performance and interpretability. Unlike traditional black-box models, the proposed method clearly reveals the major process and feedstock variables that control the variation in bio-oil yield. In practice, this knowledge can support better decisions, minimize trial-and-error experiments, improve process control, and enable more efficient design and scale-up of biomass pyrolysis systems, which in turn will lead to better operational reliability and more efficient use of resources. The research implements a well-structured workflow to deliver accurate, interpretable bio-oil yield predictions effectively. Initially, the physicochemical properties of biomass, as well as pyrolysis operating conditions and experimental data gathered from the literature, are preprocessed through normalization. After that, feature screening and selection are employed to identify relevant inputs, remove redundancies, and enhance the model’s robustness. Several machine learning regression models are built and compared using the training, validation, and testing datasets. Metaheuristic optimization algorithms are combined to further improve the models by carrying out optimal hyperparameter tuning. To determine feature importance and explain model output, global sensitivity analysis and SHAP-based interpretability techniques are implemented. Finally, prediction uncertainty is assessed with prediction intervals, and the outcomes are converted into decision support tools for feedstock selection, process optimization, and risk-aware operation (Fig. 1).

## Data Collection

The dataset for bio-oil yield analysis was mainly sourced from existing studies and comprises 245 entries. Each record is a complete set of relevant variables, including results of ultimate and proximate analyses, as well as the corresponding pyrolysis operating conditions. In order to avoid the effect of the aqueous phase, all the reported bio-oil yields were converted to a dry basis of the organic phase. The data set includes a variety of biomass feedstocks such as herbaceous materials, lignocellulosic biomass, and algae. Such a range allows for a thorough investigation of the effects of biomass physicochemical characteristics and pyrolysis conditions on the yield and quality of the bio-oil produced.

One should be aware that the dataset has two unavoidable sources of uncertainty. Initially, measurement-related discrepancies originate because some papers indirectly determine the fixed carbon content by difference. Additionally, differences in experimental setups and equipment across laboratories introduce extra experimental uncertainty. Once the dataset was ready, the Pearson correlation coefficient (PCC) was used to assess the linear relationships among certain variables. To reduce the impact of variations in parameter scales on the analytical results, all variables were normalized before the next analysis. Normalization was done with the Standard Scaler function from the scikit-learn library, which normalizes features by first removing the mean and then dividing by the standard deviation^21^.

The dataset was compiled from previously published experimental studies that reported complete biomass characterization and pyrolysis operating conditions^21^. Although the collected samples represent a broad range of biomass categories, including herbaceous biomass, lignocellulosic materials, and algae, detailed metadata describing the geographic origin of individual feedstocks, publication period, and reactor configuration were not consistently available across the sources. Consequently, these factors were not included as predictive variables in the present study. Variations in reactor design, such as fixed-bed, fluidized-bed, auger, or other laboratory-scale pyrolysis systems, together with differences in heating characteristics and operational protocols, may contribute to additional variability beyond the measured biomass properties and operating conditions. Therefore, the developed model should be interpreted as a generalized predictive framework based on the available physicochemical and process variables rather than a reactor-specific prediction model.

Figure 2 shows a detailed data summary of the input variables (Ash, FC, VC, C, H, N, O, and T) and the corresponding Yield target using polar scatter plots. Each feature is represented across a complete 0–360° angular range, thus allowing the evaluation of uniformity in direction, variation in radius, and density patterns. The fairly uniform angular spread in all the charts suggests that the data were equally sampled and there is no directional bias in the dataset. The differences in radial extent indicate variability in the features, with FC, C, and H having a wider spread and hence likely higher intrinsic variability, whereas T (°C) and Yield have narrower distributions that correspond with more stable behavior. The Yield distribution, in particular, is consistent in structure with several input features, indicating possible nonlinear relationships and interaction effects.

Fig. 2Feature-based data summary showing the distribution characteristics of input variables and the corresponding Yield target.

**Figure:**
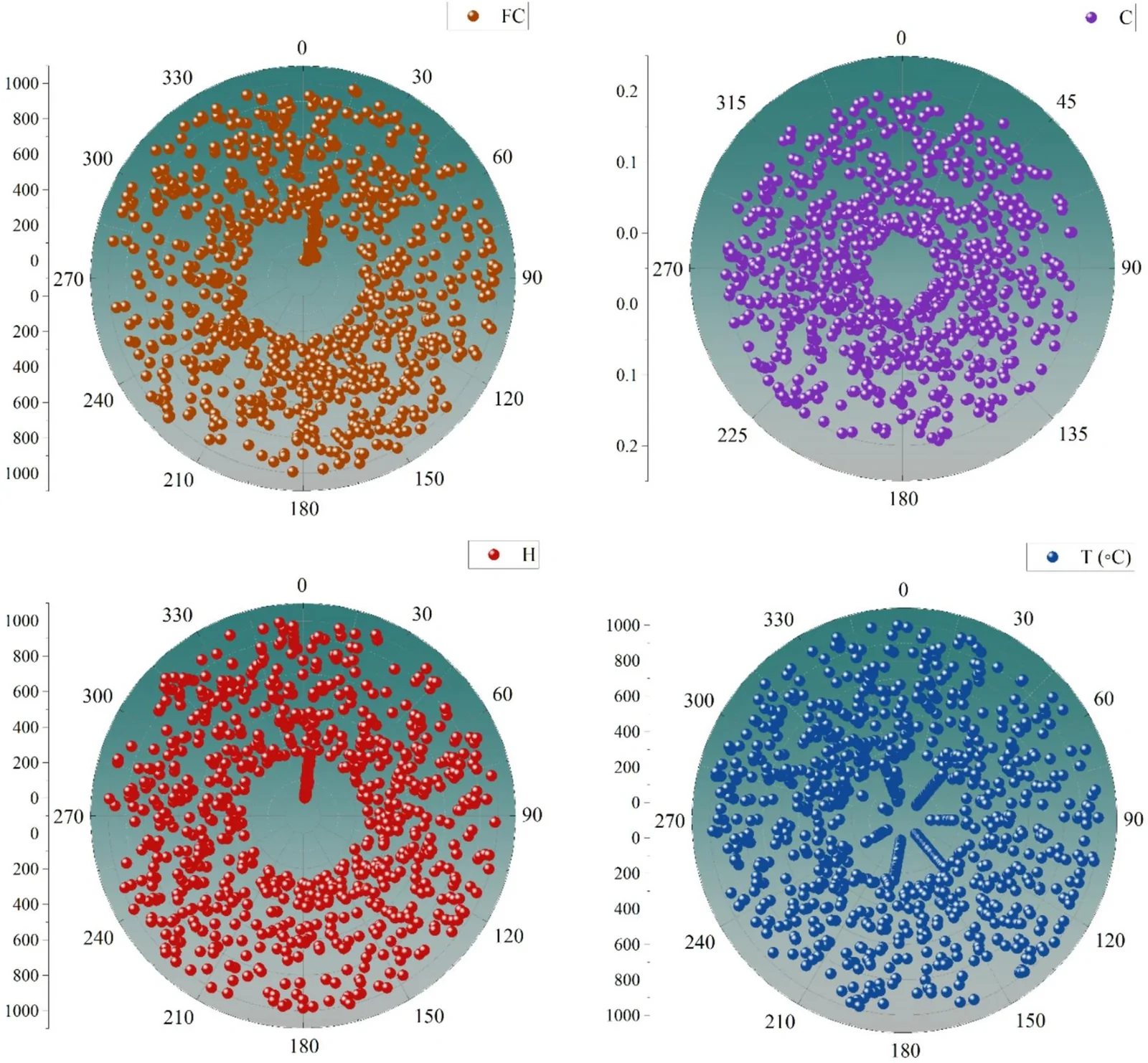

**Figure:**
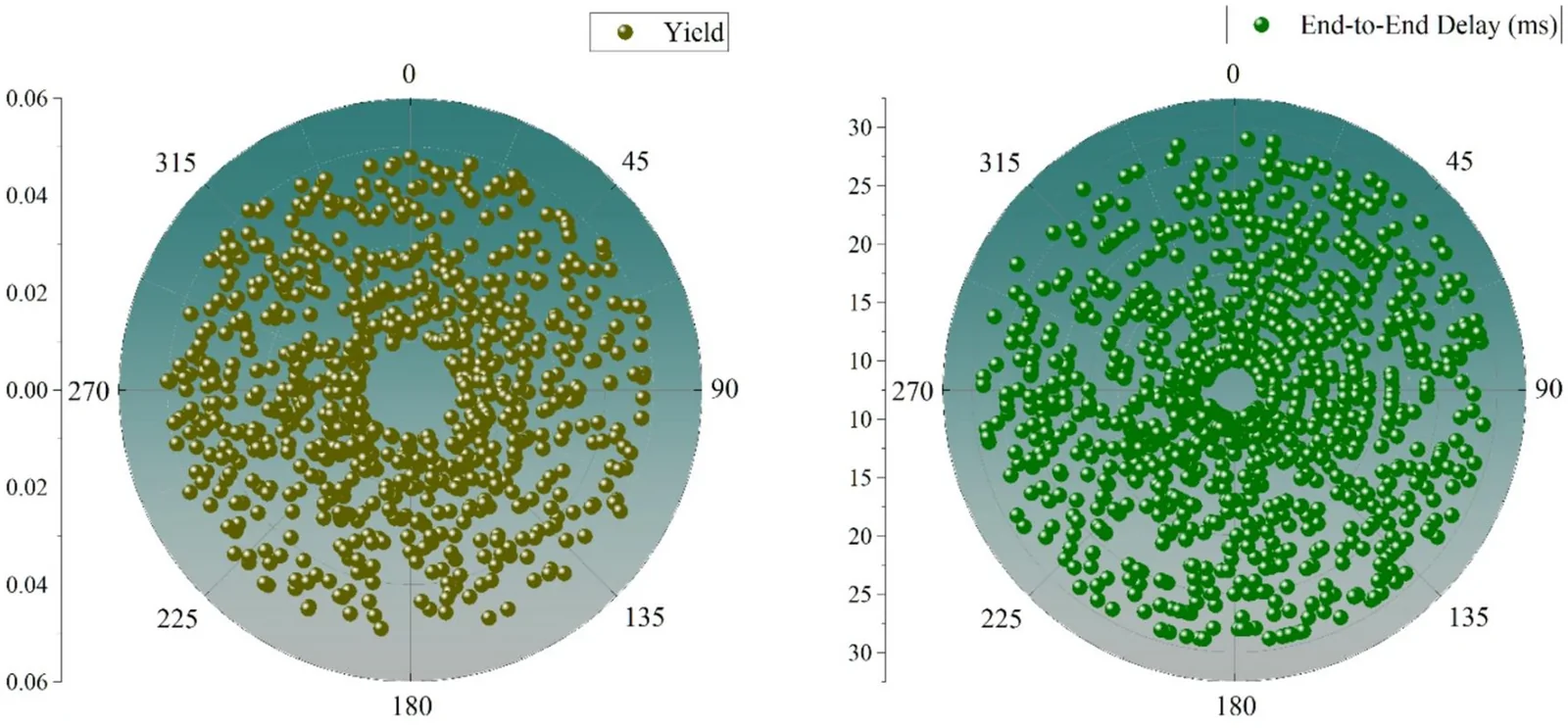


Table 1 shows the statistical breakdown of the feature variables and the target output (Yield) used for predictive modeling; thus, the table provides a detailed quantitative description of the data ranges and variability. Temperature, T, ranges from 300 to 700 °C and thus defines the boundary condition of the experiments. The statistical summary indicates that the input variables exhibit different ranges and levels of variability, reflecting the heterogeneous physicochemical characteristics of the biomass feedstocks and pyrolysis operating conditions. To reduce the influence of differences in feature scales, all variables were standardized using the StandardScaler function prior to model development. Proximate and ultimate analysis components Ash, FC, VC, C, H, N, and O have different ranges and standard deviations; thus. they represent different physicochemical properties. The Volatile content (VC) and oxygen (O) have a relatively high level of variability, whereas hydrogen (H) displays a less variable pattern, which means that it is more stable. Fixed carbon (FC) and carbon (C) show moderate scatter; thus, these two elements contribute fairly equally to system behavior. Yield output is between 16.049 and 53.074 with a standard deviation of 8.260, which means there is enough variability for the model to be well trained.

**Table 1:** Statistical characterization of feature variables and target output (yield) for predictive modeling.

Distribution	Variable	Indicator			
		Upper bound		Lower bound	
Uniform	T (°C)	700		300	
		Max.	Min.	Avg.	St. Dev.
Normal	Ash	17.330	0.240	5.426	2.969
	FC	31.130	3.220	14.831	4.651
	VC	87.040	51.450	77.120	7.241
	C	60.460	39.340	50.589	5.822
	H	9.080	5.000	6.529	1.134
	N	9.290	0.220	3.738	3.284
	O	53.300	27.360	39.078	8.314
	Yield	53.074	16.049	31.342	8.260

### Feature screening and selection

To identify the most informative variables for bio-oil yield prediction and reduce unnecessary complexity, a filter-based feature screening strategy was applied. Unlike wrapper or embedded feature selection methods, which require repeated model training, the adopted approach evaluates the intrinsic statistical relationships among variables before model development. Pearson correlation coefficient (PCC) was employed to quantify the linear association between each input feature and the target bio-oil yield, while also examining potential redundancy among predictor variables. The PCC value between two variables $\:\boldsymbol{x}$ and $\:\boldsymbol{y}$ was calculated as:

1 $$\:{r}_{xy}=\frac{\sum\:_{i=1}^{n}\left({x}_{i}-\stackrel{-}{x}\right)\left({y}_{i}-\stackrel{-}{y}\right)}{\sqrt{{\sum\:_{i=1}^{n}\left({x}_{i}-\stackrel{-}{x}\right)}^{2}}\sqrt{{\sum\:_{i=1}^{n}\left({y}_{i}-\stackrel{-}{y}\right)}^{2}}}$$

where $\:{r}_{xy}$ represents the correlation coefficient, $\:{x}_{i}$ and $\:{y}_{i}$ are individual observations, and $\:\stackrel{-}{x}$ and $\:\stackrel{-}{y}$ denote the corresponding mean values. Features exhibiting meaningful relationships with bio-oil yield were retained for predictive modeling, whereas variables providing limited independent information or showing strong redundancy with other predictors were excluded. Based on this screening process, the original set of eight input variables (Ash, FC, VC, C, H, N, O, and T) was evaluated, and the selected variables were subsequently used as model inputs.

PCC-based screening was selected because it provides a transparent and physically interpretable feature-reduction mechanism, consistent with developing a feature-centric and explainable prediction framework. After feature selection, the retained variables were normalized using the StandardScaler function from the scikit-learn library to ensure comparable numerical scales among different input parameters. Among the initial eight candidate features, seven variables were retained, while one variable (N) was removed due to weak correlation with bio-oil yield.

## Methods

### Deep neural network (DNN)

A DNN^22^ is a complex neural network with numerous hidden layers, making it capable of representing complex patterns in data. It is especially adept at image classification, speech recognition, and natural language understanding through learning hierarchical feature representations. DNNs require huge computational resources and large datasets to train and thus are best suited for unstructured data. They have deep architectures that enable them to learn complex non-linear relationships, but they can be affected by overfitting if not regularized and tuned.

### Light gradient boosting regression (LGBR)

LightGBM is an effective gradient-boosting system tailored for large-scale datasets and computing efficiency^23^. In contrast to XGBoost’s level-wise tree construction, LightGBM employs a leaf-wise technique with maximum depth limits, enabling the formation of deeper trees with fewer splits, hence enhancing accuracy. This approach promotes the leaf that most effectively minimizes loss, resulting in accelerated convergence and enhanced generalization. LightGBM utilizes a histogram-based approach to enhance memory efficiency and processing speed by discretizing continuous features into k bins, converting floating-point values into compact 8-bit representations. This substantially decreases memory consumption to one-eighth of the initial size without compromising accuracy, attributable to the regularization impact of coarser divisions. Model training seeks to minimize the loss function throughout the dataset $\:X={\left\{{x}_{i},{y}_{i}\right\}}_{i=1}^{m}$

2 $$\:\widehat{f}\left(x\right)arg\:min{E}_{y,x}L(y,f(x\left)\right)$$

The model is constructed by progressively including regression trees:

3 $$\:{f}_{T}\left(X\right)={\sum\:}_{t=1}^{T}{f}_{t}\left(X\right)$$

At iteration t, the objective function is estimated using Newton’s approach as follows:

4 $$\:{{\Gamma\:}}_{t}\cong\:{\sum\:}_{j=1}^{N}L({y}_{i},{F}_{t-1}\left({x}_{i}\right)+{f}_{t}({x}_{i}\left)\right)$$

where $\:{g}_{i}$and $\:{h}_{i}$ denote the first- and second-order gradients, respectively. The ideal weight for a leaf node is obtained in the following manner:

5 $$\:{{\Gamma\:}}_{t}\cong\:{\sum\:}_{j=1}^{N}({g}_{i}{f}_{t}\left({x}_{i}\right)+\frac{1}{2}{h}_{i}{f}_{t}^{2}({x}_{i}\left)\right)$$

**Fig. 3:**
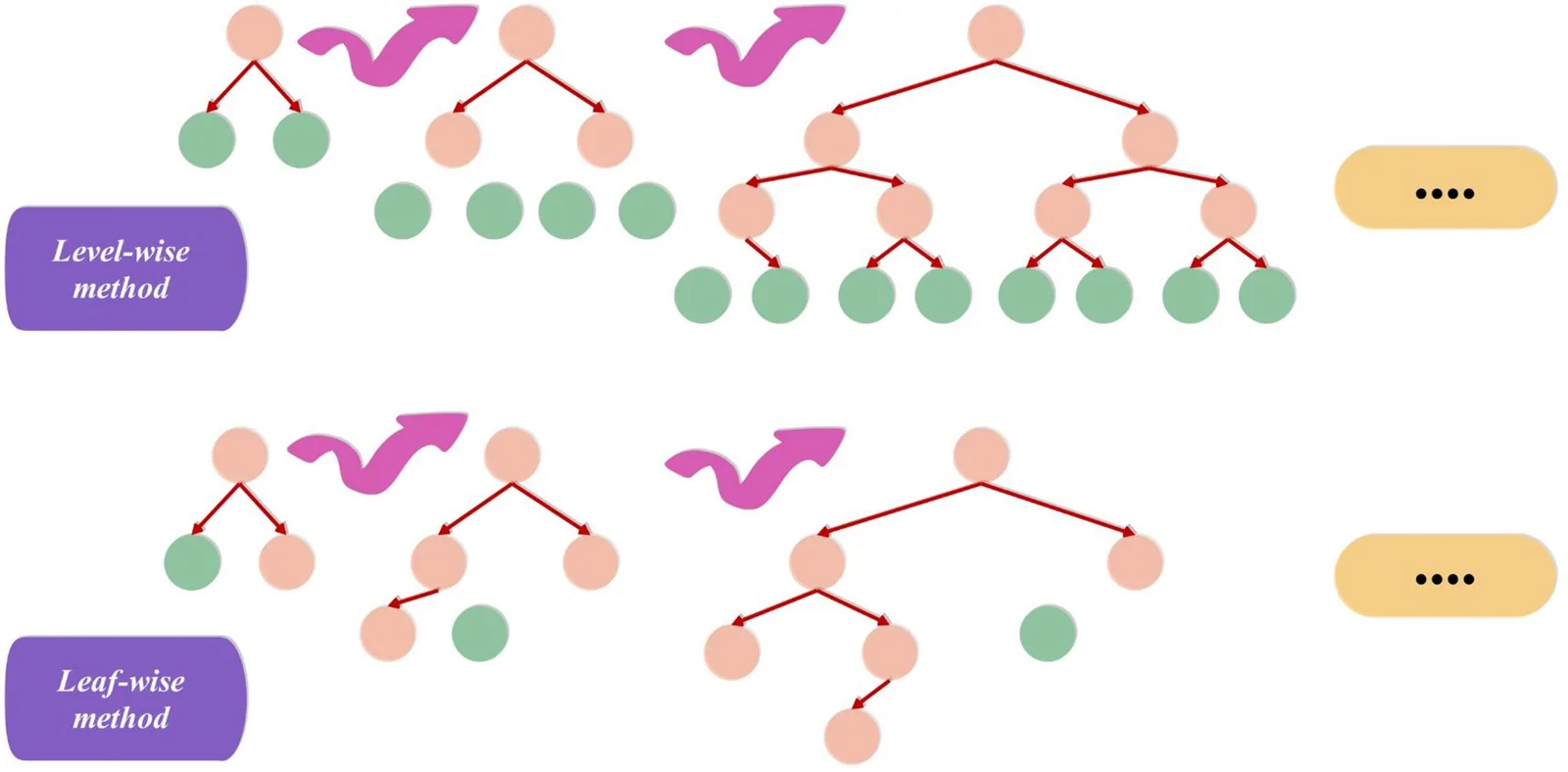
The flowchart of LGBR.

This gradient-based optimization enables LightGBM to achieve faster training and improved accuracy, particularly for unbalanced and high-dimensional datasets like Android malware traffic. Figure 3 illustrates the flowchart of the LGBR, emphasizing its histogram-based decision tree learning, leaf-wise growth approach, and effective feature management system.

### Giant armadillo optimization (GAO)

#### Mathematical modeling of GAO

This section outlines the steps of GAO implementation. The GAO launch procedure has been delineated and simulated for this particular purpose. The process of enhancing prospective solutions through two phases, exploration and exploitation, is then expressed mathematically^24^.

#### Algorithm initialization

The population-based meta-heuristic algorithm, referred to as the proposed GAO approach, is based on the premise that gigantic armadillos constitute its population. Due to its members’ robust search skills in the problem-solving arena, GAO can provide suitable solutions for optimization difficulties via a continually developing process. Each GAO member selects the settings for the problem’s characteristics based on their position within the problem-solving space. Thus, each enormous armadillo in the crowd signifies a potential solution to the problem depicted by a vector inside the mathematical framework. The demography of gigantic armadillos can be mathematically represented as a graph according to Eq. (6). Initially, the placement of the colossal armadillos is dynamically determined in the troubleshooting space using Eq. (7).

6 $$\:X={\left[\begin{array}{c}\begin{array}{c}{X}_{1}\\\:\vdots\end{array}\\\:{X}_{i}\\\:\begin{array}{c}\vdots\\\:{X}_{N}\end{array}\end{array}\right]}_{N\times\:m}={\left[\begin{array}{c}{x}_{\mathrm{1,1}}\\\:\vdots\\\:\begin{array}{c}{x}_{i,1}\\\:\vdots\\\:{x}_{N,1}\end{array}\end{array}\begin{array}{c}\dots\:\\\:\ddots\:\\\:\begin{array}{c}\dots\:\\\:{\mathinner{\mkern2mu\raise1pt\hbox{.}\mkern2mu\raise4pt\hbox{.}\mkern2mu\raise7pt\hbox{.}\mkern1mu}}\\\:\cdots\:\end{array}\end{array}\begin{array}{c}{x}_{1,d}\\\:\vdots\\\:\begin{array}{c}{x}_{i,d}\\\:\vdots\\\:{x}_{N,d}\end{array}\end{array}\begin{array}{c}\cdots\:\\\:{\mathinner{\mkern2mu\raise1pt\hbox{.}\mkern2mu\raise4pt\hbox{.}\mkern2mu\raise7pt\hbox{.}\mkern1mu}}\\\:\begin{array}{c}\cdots\:\\\:\ddots\:\\\:\cdots\:\end{array}\end{array}\begin{array}{c}{x}_{1,m}\\\:\vdots\\\:\begin{array}{c}{x}_{i,m}\\\:\vdots\\\:{x}_{N,m}\end{array}\end{array}\right]}_{N\times\:m}$$

7 $$\:{x}_{i,d}={lb}_{d}+r\times\:(u{b}_{d}-l{b}_{d})$$

In this context, * m* denotes the number of selection criteria, * N* signifies the entire population of large armadillos, and * r* is an arbitrary value within the range of 0 to 1. $\:l{b}_{d}$ and $\:u{b}_{d}$ indicate the minimum and maximum bounds of the * dth* decision parameter, respectively. The GAO demographic matrix * X*, proposed solution * Xi*, and its * dth* observations inside the search zone (decision parameter) are denoted by $\:{x}_{i,d}$^25^.

8 $$\:F={\left[\begin{array}{c}\begin{array}{c}{F}_{1}\\\:\vdots\end{array}\\\:{F}_{i}\\\:\begin{array}{c}\vdots\\\:{F}_{N}\end{array}\end{array}\right]}_{N\times\:1}={\left[\begin{array}{c}\begin{array}{c}F\left({X}_{1}\right)\\\:\vdots\end{array}\\\:F\left({X}_{i}\right)\\\:\begin{array}{c}\vdots\\\:F\left({X}_{N}\right)\end{array}\end{array}\right]}_{N\times\:1}$$

The predicted target value based on the * ith* GAO constituent is marked as $\:{F}_{i}$, whereas the array of estimated target functions is represented as * F*.

The evaluated objective function values provide significant insights into the quality of the proposed solutions submitted by the participants. The most advantageous participant, or the optimal value derived from the objective function, signifies the ideal candidate solution. In contrast, the least favorable participant, or the suboptimal candidate solution, is indicated by the objective function’s minimum value. Due to the shifting placement of the gigantic armadillos in the problem-solving region with each iteration, the most favorable member must be adjusted based on the objective function’s comparative values. As the algorithm develops, the location of the top member, determined by its cycles, is presented as the solution to the issue.

The proposed GAO approach alters the positioning of participants inside the problem-solving domain, emulating the foraging strategy of large armadillos in their habitat. The colossal armadillo attacks termite mounds during this process before burrowing into them to locate and consume termites. The relative positions of the group of participants have been revised for each GAO iteration in two stages: (i) exploration, modeled on giant armadillos approaching termite mounds; (ii) exploitation, based on massive armadillos excavating termite mounds.

#### First stage: exploration and attack on termite mounds

In the preliminary phase of GAO, residents in the region facing addressing issues are relocated according to a model inspired by the giant armadillo’s predation on termite nests during its hunting season. The colossal armadillo’s changing posture when nearing the termite mounds inspires the GAO design, which revises the positioning of population members inside the problem-solving domain. Replicating this attack strategy yields significant variability in the placement of the giant armadillo, augmenting the algorithm’s exploratory capacity for worldwide surveillance.

The positioning of everyone else with a superior objective function value is termed an insect nesting in the GAO design, which illustrates a colossal armadillo for each member of the general public. Equation (9) delineates the set of potential termite nests for each member within the population.

9 $$\:T{M}_{i}=\left\{{X}_{k}:{F}_{k}<{F}_{i}\:and\:k\ne\:i\right\},\:where\:i=\mathrm{1,2},\dots\:,N\:and\:k\in\:\{\mathrm{1,2},\dots\:,N\}$$

In this instance, the objective function value of the * ith* giant armadillo is denoted by $\:{F}_{k}$, which is the population member with a superior objective function value compared to $\:{X}_{k}$, and the collection of prospective termite pile locations for it is denoted by $\:T{M}_{i}$.

The giant armadillo arbitrarily selects one of the probable termite mounds and thereafter attacks it. Equation (10) is employed to ascertain the new position of each population member based on a model of giant armadillo migration towards termite mounds. If this new location, as per Eq. (11), improves the value of the objective function, it will replace the previous position of the corresponding member.

10 $$\:{x}_{i,j}^{P1}={x}_{i.j}+{r}_{i,j}\times\:\left({STM}_{i.j}-{I}_{i,j}\times\:{x}_{i.j}\right),$$

11 $$\:{X}_{i}=\left\{\begin{array}{c}{X}_{i}^{P1},{F}_{i}^{P1}\le\:{F}_{i},\\\:{X}_{i},\:\:else,\end{array}\right.$$

In this context, $\:ST{M}_{i}$ represents the selected termite mound of the enormous armadillo, whereas $\:ST{M}_{i.j}$ denotes its * jth* dimension. The new position of the * ith* gigantic armadillo, $\:{x}_{i}^{P1}$, is established by the assault stage of the proposed GAO. $\:{F}_{i}^{P1}$ represents the evaluation of its objective function, whereas $\:{x}_{i,j}^{P1}$ denotes its * jth* dimension. $\:{I}_{i,j}$ are numbers randomly selected to be either 1 or 2, whereas $\:{r}_{i,j}$ are random values within the interval $\:\left[\mathrm{0,1}\right]$.

#### Second stage of termite mound exploitation; excavation

The simulated scenario of a giant armadillo excavating termite mounds for sustenance has enhanced the identification of persons addressing concerns during the second phase of GAO. In simulating the gigantic armadillo’s foraging activity to find and devour termites, the armadillo’s position is slightly modified, enhancing the algorithm’s capacity for local search optimization^26^.

In the GAO design, the new location for each population member is determined using Eq. (12), which models the ability of the immense armadillo to excavate termite mounds. Consequently, according to Eq. (13), this new position replaces the individual’s previous placement if the objective function value improves.

12 $$\:{x}_{i,j}^{P2}={x}_{i.j}+\left(1-2{r}_{i,j}\right)\times\:\frac{u{b}_{j}-l{b}_{j}}{t}$$

13 $$\:{X}_{i}=\left\{\begin{array}{c}{X}_{i}^{P2},{F}_{i}^{P2}\le\:{F}_{i}\\\:{X}_{i},\:\:else\end{array}\right.$$

$\:{X}_{i}^{P2}$ represents the new position established by the planned GAO’s excavation phase for the * ith* enormous armadillo. $\:{F}_{i}^{P2}$ represents the value of its objective function, $\:{x}_{i,j}^{P2}$ represents its * jth* dimension, whereas $\:{r}_{i,j}$ denotes numbers randomly chosen from the range of $\:\left[\mathrm{0,1}\right]$, with * t* functioning as the repetition counter.

### Mother optimization algorithm (MOA)

The MOA is a single iterative, population-based metaheuristic that efficiently performs optimization^27^. Each population member represents a possible solution (candidate) represented by a vector of decision variables in the search space.

First, the algorithm sets up the population matrix * X* with the help of Eqs. (14)–(15):

14 $$\:X={\left[\begin{array}{c}{X}_{1}\\\:\vdots\\\:\begin{array}{c}{X}_{i}\\\:\vdots\\\:{X}_{n}\end{array}\end{array}\right]}_{n\times\:d}={\left[\begin{array}{ccc}{x}_{\mathrm{1,1}}&\:\cdots\:&\:{x}_{1,j}\\\:\vdots&\:\ddots\:&\:\vdots\\\:\begin{array}{c}{x}_{i,1}\\\:\begin{array}{c}\vdots\\\:{x}_{n,1}\end{array}\end{array}&\:\begin{array}{c}\cdots\:\\\:\begin{array}{c}{\mathinner{\mkern2mu\raise1pt\hbox{.}\mkern2mu\raise4pt\hbox{.}\mkern2mu\raise7pt\hbox{.}\mkern1mu}}\\\:\cdots\:\end{array}\end{array}&\:\begin{array}{c}{x}_{i,j}\\\:\begin{array}{c}\vdots\\\:{x}_{n,j}\end{array}\end{array}\end{array}\begin{array}{cc}\:\:\:\:\:\begin{array}{c}\cdots\:\\\:{\mathinner{\mkern2mu\raise1pt\hbox{.}\mkern2mu\raise4pt\hbox{.}\mkern2mu\raise7pt\hbox{.}\mkern1mu}}\\\:\begin{array}{c}\cdots\:\\\:\begin{array}{c}\ddots\:\\\:\cdots\:\end{array}\end{array}\end{array}&\:\begin{array}{c}{x}_{1,d}\\\:\vdots\\\:\begin{array}{c}{x}_{i,d}\\\:\begin{array}{c}\vdots\\\:{x}_{n,d}\end{array}\end{array}\end{array}\end{array}\right]}_{n\times\:d}$$

15 $$\:{x}_{i,j}={lb}_{j}+\mathcal{r}\left(\mathrm{0,1}\right).\left({Ub}_{j}-{Lb}_{j}\right),\:\:i=\mathrm{1,2},\dots\:,n,\:\:j=\mathrm{1,2},\dots\:,\:d.$$

where * n* stands for population size, * d* represents the number of decision variables, and and are the lower and upper bounds of the th variable, respectively. The term $\:\mathcal{r}\left(\mathrm{0,1}\right)$ indicates a uniform random number generation in the range of $\:\left[\mathrm{0,1}\right]$. Thus, a single individual $\:{X}_{i}=\left({x}_{i,1},\:\dots\:,{x}_{i,j},\:\dots\:,{x}_{i,d}\right)$ in the search space represents a candidate solution. The objective function is computed for each individual by using Eq.‌(15):

16 $$\:P={\left[\begin{array}{c}{P}_{1}\\\:\vdots\\\:\begin{array}{c}{P}_{i}\\\:\vdots\\\:{P}_{n}\end{array}\end{array}\right]}_{n\times\:1}={\left[\begin{array}{c}{P(X}_{1})\\\:\vdots\\\:\begin{array}{c}{P(X}_{i})\\\:\vdots\\\:{P(X}_{n})\end{array}\end{array}\right]}_{n\times\:1}$$

where‍‌ $\:{P}_{i}$ is the value of the objective function (fitness) of the * i*th candidate. The individuals with the best and worst performances are identified based on their objective function values, and the best one (the mother) leads the population through three main stages metaphorically derived from mother-child interactions:

Phase 1: Exploration phase (education).

The stage here simulates the learning process when the mother teaches her kids, thereby improving the algorithm’s global search capability. The position of every individual is changed in the following ‍way:

17 $$\:{{x}_{i,j}}^{v1}={x}_{i,j}+\mathcal{r}\left(\mathrm{0,1}\right).\left({M}_{j}-\mathcal{r}\left(2\right).{x}_{i,j}\right),$$

18 $$\:{X}_{i}=\left\{\begin{array}{c}{{X}_{i}}^{v1},\:\:{{P}_{i}}^{v1}\le\:{P}_{i},\\\:{X}_{i},\:\:\:\:\:\:\:\:\:\:\:\:\:\:else,\:\:\:\:\:\:\:\:\end{array}\right.$$

Here $\:{x}_{i,j}$ denotes the * j*th feature of the * i*th element, $\:{M}_{j}$ represents the * j*th feature of the best individual (mother), $\:\mathcal{r}\left(\mathrm{0,1}\right)$ is a random number in $\:\left[\mathrm{0,1}\right]$, and $\:\mathcal{r}\left(2\right)$is a random selection from $\:\left\{\mathrm{1,2}\right\}$. $\:{{x}_{i,j}}^{v1}$ and $\:{{P}_{i}}^{v1}$ are the new coordinates and the new value of the objective function of the * i*th member after this step, respectively.

Phase 2: Exploration phase (advice).

During this phase, the mother gives some advice to stop the misbehavior. Those members who have the worst fitness values are adjusted by looking at those who have better performance:

19 $$\:{BB}_{i}=\left\{{X}_{k},\:{P}_{k}>{P}_{i}\wedge\:k\in\:\left\{\mathrm{1,2},\dots\:,\:n\right\}\right\},\:\:where\:i=\mathrm{1,2},\dots\:,\:n$$

20 $$\:{{x}_{i,j}}^{v2}={x}_{i,j}+\mathcal{r}\left(\mathrm{0,1}\right).\left({x}_{i,j}-\mathcal{r}\left(2\right).{SBB}_{i,j}\right)$$

21 $$\:{X}_{i}=\left\{\begin{array}{c}{{x}_{i}}^{v2},\:\:{{P}_{i}}^{v2}\le\:{P}_{i}\:\\\:{X}_{i},\:\:\:\:\:\:\:\:\:\:\:\:\:else,\:\:\:\:\:\:\:\:\:\end{array}\right.$$

$\:{BB}_{i}$‍‌‍‍‌ stands for the “bad behaviors” (individuals with higher objective values). $\:{SBB}_{i}$ is a randomly chosen bad behavior from $\:{BB}_{i}$, and $\:{SBB}_{i,j}$ is its * j*th dimension. $\:{{x}_{i,j}}^{v2}$ denotes the newly updated position, and $\:{{P}_{i}}^{v2}$ is the corresponding fitness value. This operation expands the search area, preventing the algorithm from being trapped in local minima.

Phase 3: Exploitation phase (upbringing).

This upbringing stage locally refines solutions to improve convergence by introducing small, controlled modifications:

22 $$\:{{x}_{i,j}}^{v3}={x}_{i,j}+\left(1-2.\mathcal{r}\left(\mathrm{0,1}\right)\right).\frac{{Ub}_{j}-{Lb}_{j}}{t},$$

23 $$\:{X}_{i}=\left\{\begin{array}{c}{{x}_{i}}^{v3},\:\:{{P}_{i}}^{v3}\le\:{P}_{i}\:\\\:{X}_{i},\:\:\:\:\:\:\:\:\:\:\:\:\:else,\:\:\:\:\:\:\:\:\:\end{array}\right.$$

Here, * t* denotes the current iteration number, $\:{{P}_{i}}^{v3}$ corresponds to the new objective function value, $\:{{x}_{i,j}}^{v3}$ represents the updated variable, and $\:{{x}_{i}}^{v3}$ is the solution that goes with it. With this stage, the algorithm gets better at local search (exploitation), slowly converging to the global optimum as iterations advance.

### Performance evaluators

The coefficient of determination (R^2^) is a statistic that signifies the percentage of variation in the real data that the model captures; hence, it is a measure of how well the model fits the data. The closer the value is to one, the higher the explanatory power of the model and the better the agreement between the predicted and the observed values^28^. The root mean square error (RMSE) measures how far, on average, the predictions are from the true values. It is calculated as the square root of the average of the squared deviations. Since it is more affected by large deviations, RMSE indicates whether there are large discrepancies between the predicted and true values and is most useful when large errors are not acceptable^29^. The mean absolute percentage error (MAPE) measures the average size of errors in percentage terms, giving an easy-to-understand measure of how accurate a forecast generally is, regardless of the unit of the variable involved. It is especially convenient when comparing the accuracy of forecasting methods across two or more datasets with different scales^30^. The coefficient of variation (COV) is a measure of relative variability that shows the ratio of the standard deviation of prediction errors to the mean of the observed values. COV helps in evaluating the consistency and dependability of the model under different scenarios^31^. The mean absolute relative difference (MARD) is a statistic that measures the mean absolute percentage difference between predicted and actual values. MARD is a great measure of predictive accuracy, and unlike metrics based on squared errors, it reduces the impact of outliers^32^.

## Result and discussion

**Fig. 4:**
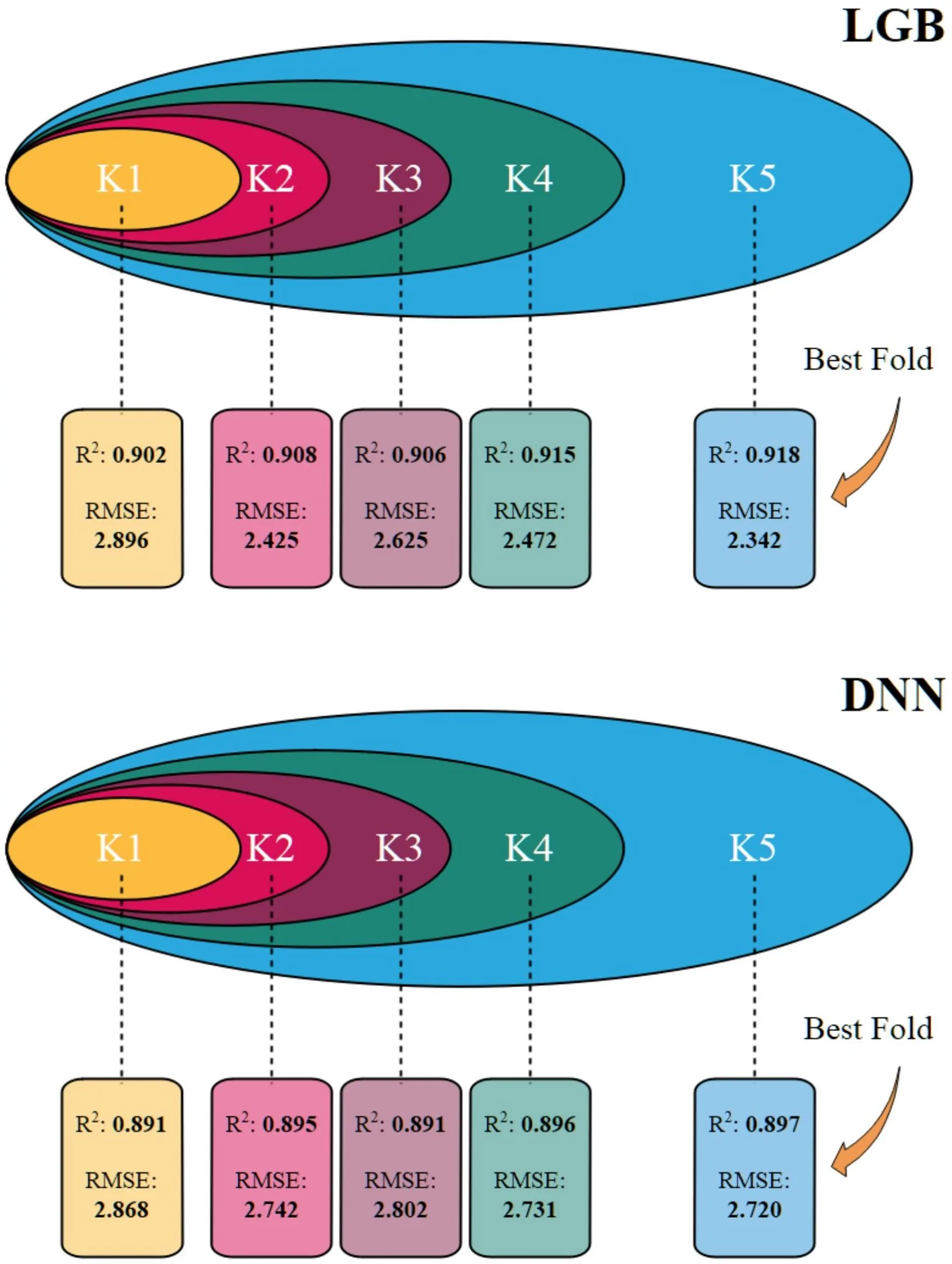
Five-fold cross-validation performance comparison of LGB and DNN models across different folds.

Figure 4 presents the fold-wise performance variation obtained from five-fold cross-validation for the LGB and DNN models. The comparison focuses on the consistency of individual folds rather than the relative ranking of the models. For the LGB model, the obtained R^2^ values across K1–K5 were 0.902, 0.908, 0.906, 0.915, and 0.918, respectively, while the corresponding RMSE values were 2.896, 2.425, 2.625, 2.472, and 2.342. Among the five folds, K5 achieved the highest predictive performance with the largest R^2^ (0.918) and the lowest RMSE (2.342), indicating the most favorable validation split. Similarly, the DNN model showed R^2^ values of 0.891, 0.895, 0.891, 0.896, and 0.897 with RMSE values of 2.868, 2.742, 2.802, 2.731, and 2.720. The best fold for DNN was also K5, providing the highest R^2^ (0.897) and lowest RMSE (2.720). Overall, K5 demonstrated the most reliable data partition for both models, reflecting stronger generalization capability compared with the remaining folds.

Table 2 most comprehensively evaluates feature-based predictive models in the training, validation, and testing phases through several performance metrics. In the training phase, hybrid models beat single models at every turn, with LGMO achieving the highest accuracy (R^2^ = 0.9555) and the lowest error (RMSE = 1.9802), thus validating the effectiveness of hybrid optimization. During the validation phase, the LightGBM-based hybrid models maintained superior predictive performance, with LGGA and LGMO achieving the highest R^2^ (0.9572 and 0.9508, respectively) while simultaneously producing the lowest prediction errors. The testing results further confirmed the robustness of the hybrid models, with DNMO and LGMO consistently outperforming their corresponding baseline models across most evaluation metrics. It should be noted that the COV represents the relative variability of the target bio-oil yield distribution within each dataset partition and is independent of the selected predictive model. Therefore, identical COV values are obtained for models evaluated on the same training, validation, or testing subset. In contrast, metrics such as R^2^, RMSE, MAPE, and MARD directly depend on model predictions and are used to quantify differences in predictive performance among the developed models. In addition, the differences between validation and testing results are attributed to the heterogeneous characteristics of the compiled biomass pyrolysis dataset. Although five-fold cross-validation was employed with an 80/20 training–evaluation partition, individual subsets may contain different combinations of feedstock properties and pyrolysis operating conditions, leading to variations in prediction difficulty. Consequently, slightly higher errors in the testing phase are expected because these samples represent independent observations not directly involved in model development. The consistent superiority of the hybrid models across evaluation phases demonstrates their robustness despite variations among data partitions.

**Table 2:** Evaluation metrics calculated for feature-based predictive models.

Phase	Category	Models	Data metrics				
			*R* ^*2*^	RMSE	MAPE	COV	MARD
Train	Single	DNN	0.8939	2.9508	5.2224	0.2722	0.0522
		LGB	0.9152	2.5675	4.3271	0.2722	0.0433
	Hybrid	DNGA	0.9270	2.4270	4.0576	0.2722	0.0406
		DNMO	0.9404	2.1350	3.5422	0.2722	0.0354
		LGGA	0.9375	2.2266	3.6857	0.2722	0.0369
		LGMO	0.9555	1.9802	3.2909	0.2722	0.0329
Validation	Single	DNN	0.8685	1.5540	2.9565	0.1207	0.0296
		LGB	0.8981	1.3610	2.5365	0.1207	0.0254
	Hybrid	DNGA	0.9249	2.0127	3.1804	0.2550	0.0318
		DNMO	0.9307	1.8977	2.8030	0.2550	0.0280
		LGGA	0.9572	0.9687	2.0167	0.1207	0.0202
		LGMO	0.9508	0.9469	1.8613	0.1207	0.0186
Test	Single	DNN	0.8338	3.0399	5.2966	0.2550	0.0530
		LGB	0.8619	2.6331	4.5513	0.2550	0.0455
	Hybrid	DNGA	0.8969	1.5742	3.3055	0.1207	0.0331
		DNMO	0.9057	1.4083	2.8492	0.1207	0.0285
		LGGA	0.9269	2.1006	3.4189	0.2550	0.0342
		LGMO	0.9165	2.0818	3.3468	0.2550	0.0335

The metaheuristic algorithms were applied to optimize the most influential hyperparameters of the developed predictive models. For the DNN-based models, the optimization variables included learning rate, number of hidden layers, neurons per hidden layer, and batch size. For the LightGBM-based models, the optimized parameters consisted of learning rate, number of leaves, number of estimators, and maximum depth. The search boundaries were predefined based on commonly adopted ranges for regression modeling and computational feasibility. Within these boundaries, the optimization algorithms explored different hyperparameter combinations to minimize prediction error, and the final selected configurations are presented in Table 3.

**Table 3:** Hyperparameter configuration of the proposed models.

Parametrs	Range	DNN	DNGA	DNMO	LGBR	LGGA	LGMO
Learning_rate	0.001–0.1	0.005	0.05	0.08	0.01	0.08	0.1
Number of hidden layers	1–5	3	4	5	–	–	–
Neurons per hidden layer	16–256	64	128	256	–	–	–
Batch size	16–128	32	64	128	–	–	–
Num_leaves	10–100	–	–	–	41	52	60
N_estimators	100–500	–	–	–	200	300	325
Max_depth	2–10	–	–	–	3	5	7

**Fig. 5:**
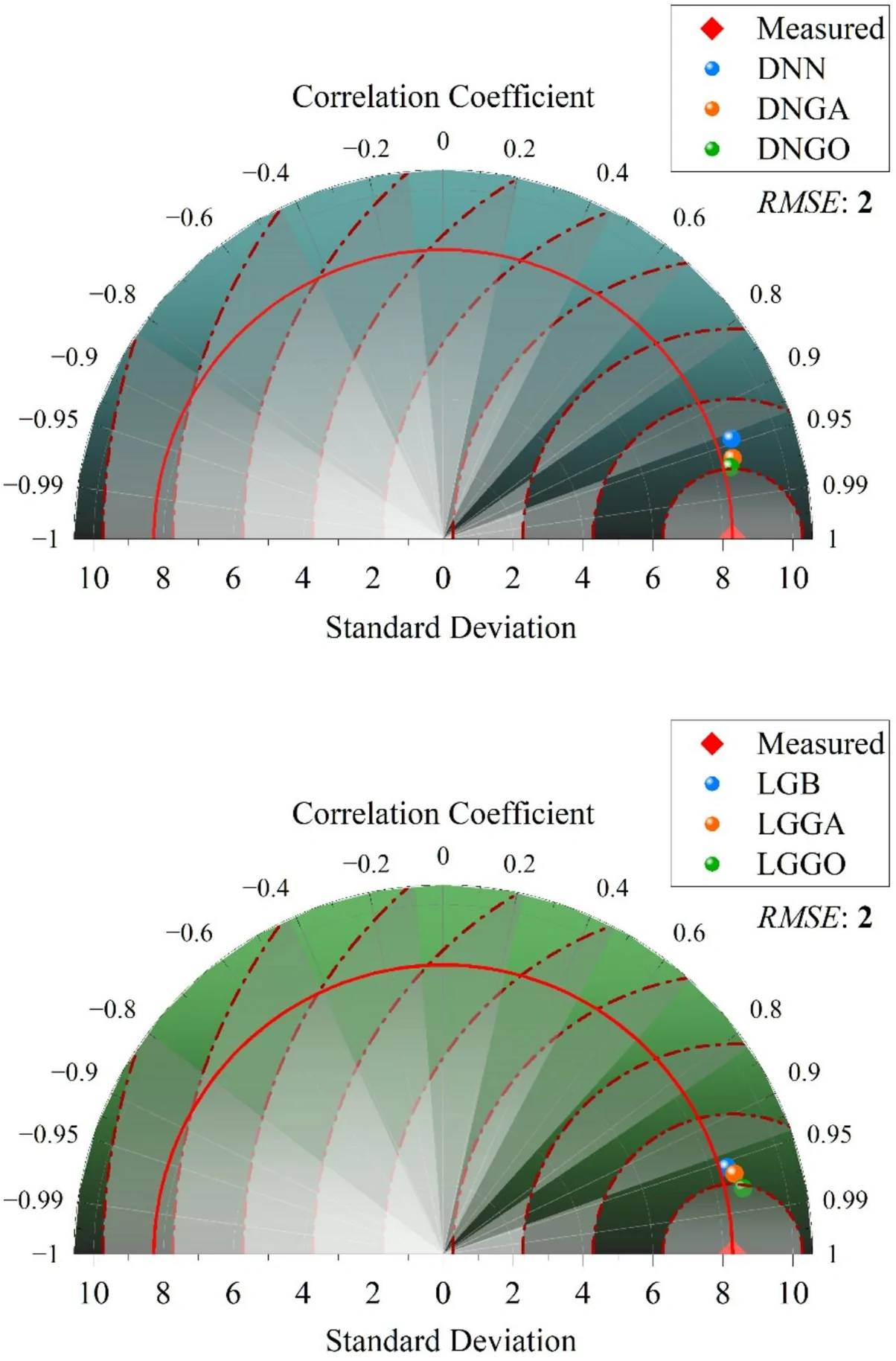
Taylor plot evaluating model predictions against measured values for the feature-based dataset using correlation, RMSE, and variability metrics.

In Fig. 5, Taylor diagrams illustrate the predictive accuracy of the proposed feature-based models compared with measured values. Three evaluation metrics—standard deviation, correlation coefficient, and RMSE—are used as an integrated set of criteria for comparison. The upper diagram is dedicated to the assessment of DNN-based models (DNN, DNGA, and DNMO), whereas the lower one refers to LGB-based models (LGB, LGGA, and LGMO). In both scenarios, the observed data are the benchmark, and predictions with higher correlation scores (closer to one), lower RMSE values, and standard deviations similar to the observed values indicate better model performance. The hybrids show a significant gain over the single models, as seen in their closeness to the benchmark. Out of all the models, LGMO best matches the measured data, as it attains the highest correlation and the smallest deviation, with LGGA and DNMO as the closest runners-up.

Figure 6 compares six models using normal probability (Q–Q) plots of feature-based prediction errors from measured values. Each subplot shows ordered residuals against theoretical normal quantiles, together with regression lines and 95% confidence envelopes; thus, distributional conformity, bias, and dispersion can be evaluated. Models whose points almost follow the reference line suggest that the model errors are more normal and thus error behavior is more reliable, whereas systematic curvature or tail deviations indicate skewness, heavy tails, or heteroscedasticity. The use of violin plots visually captures the residual density distribution, showing the central tendency, spread, and asymmetry in a succinct manner. Hence, models with narrower, more symmetric violins and smaller tail extensions tend to have better prediction stability. Variation in feature representation quality is, therefore, the main reason for differences among models, which in turn affects residual variance and tail behavior.

Fig. 6Comparison of prediction errors across models, represented through residual deviations from measured values.

**Figure:**
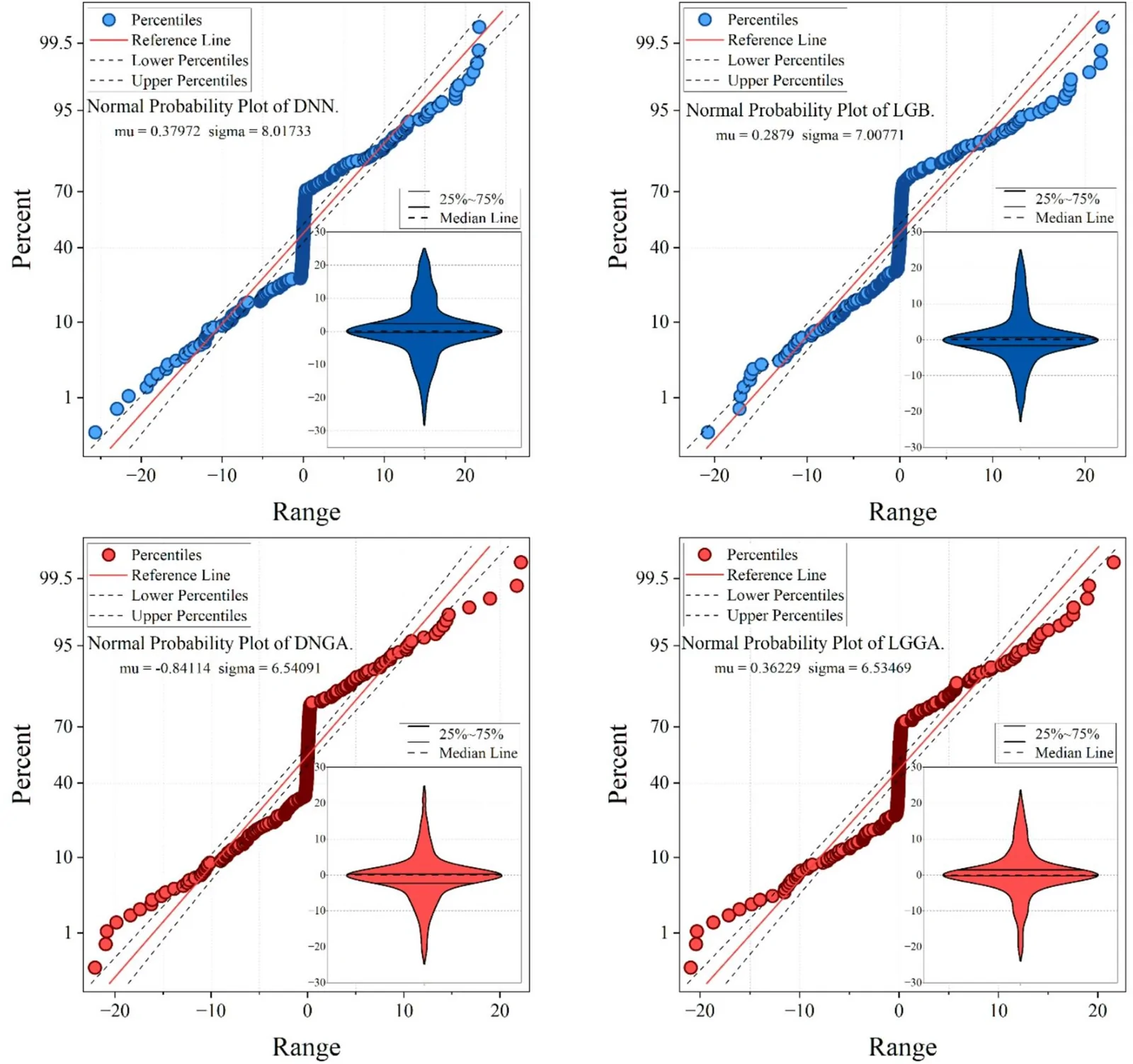

**Figure:**
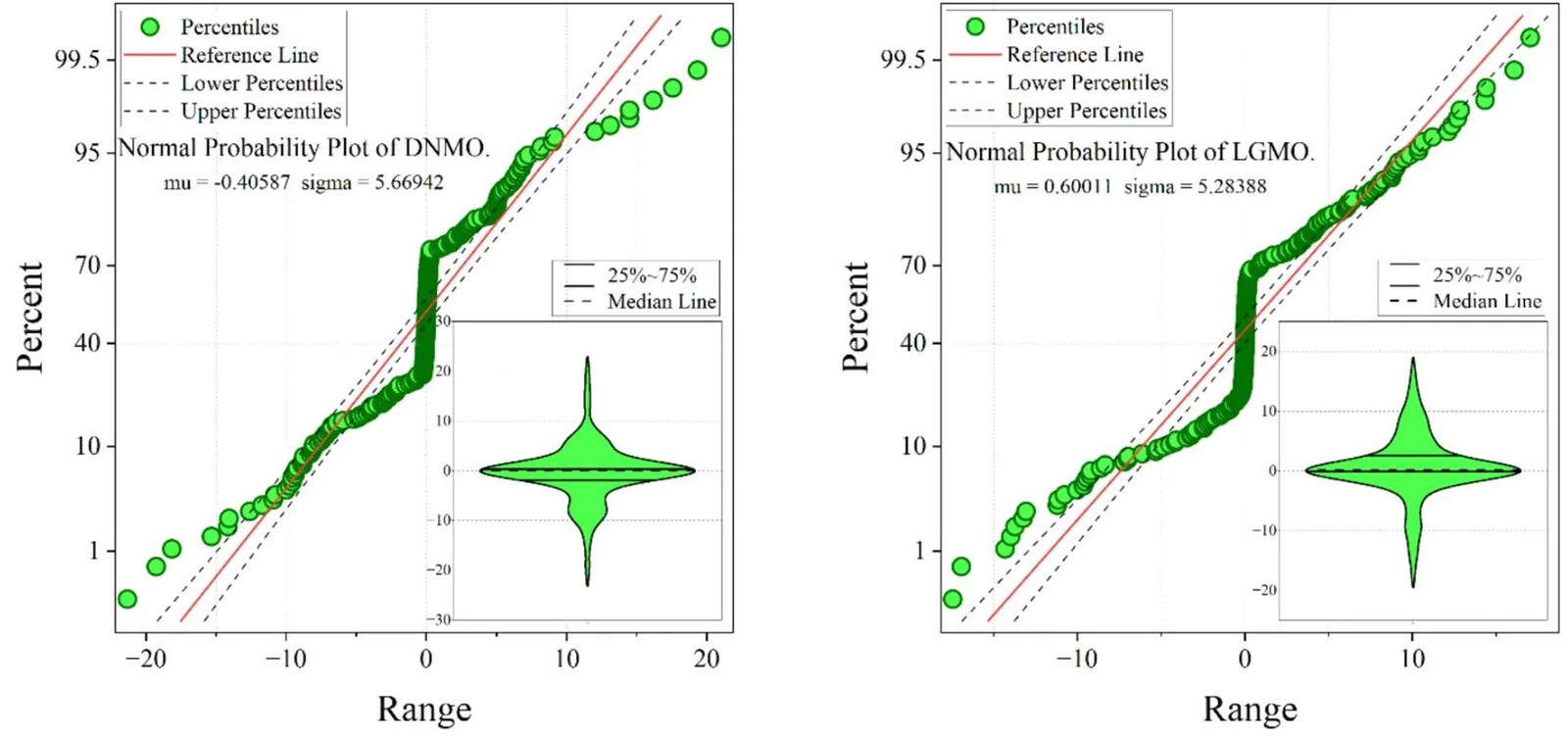


**Fig. 7:**
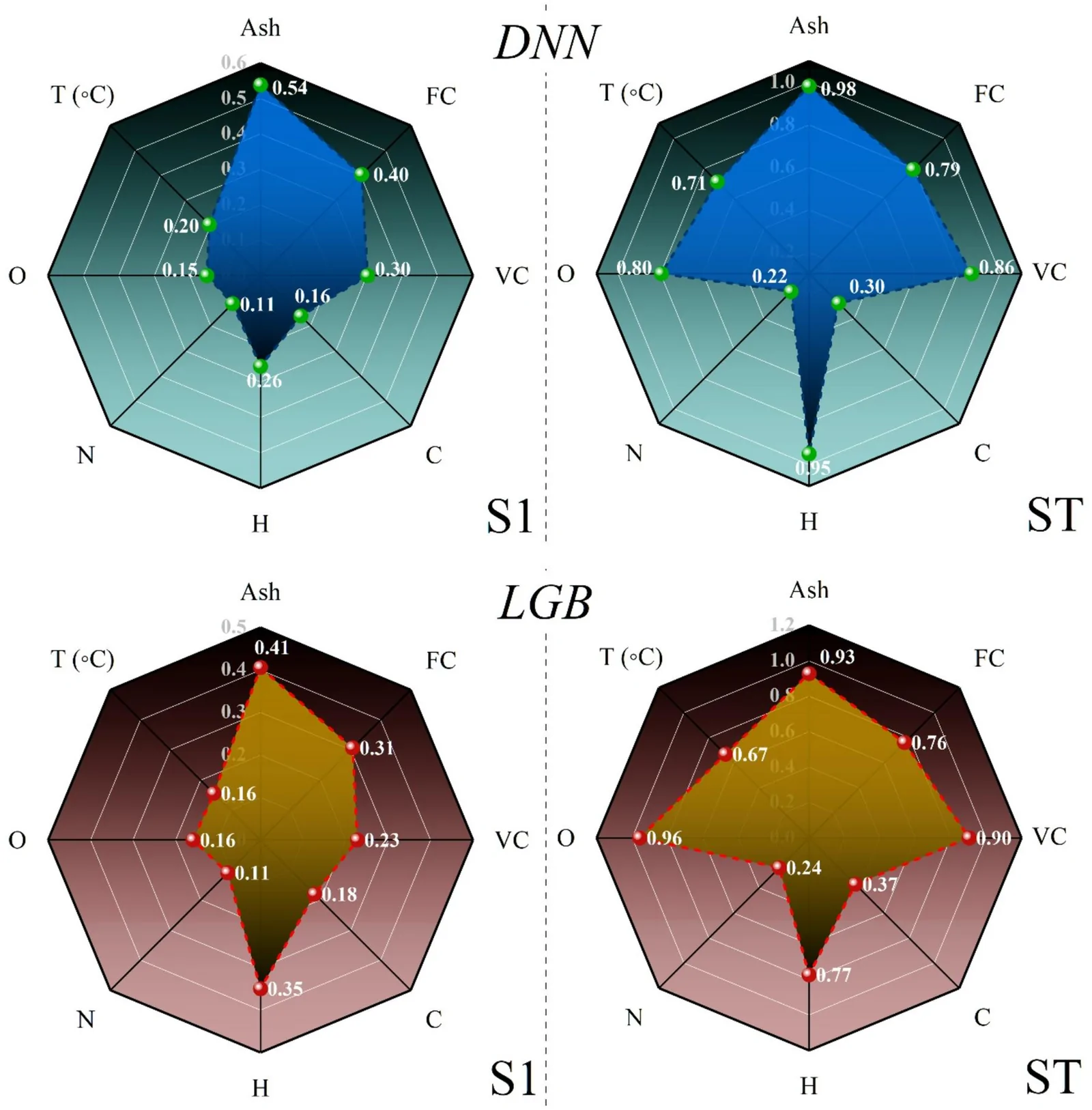
FAST-based sensitivity ranking depicting the most influential features driving variability in the model output.

Figure 7 shows how the FAST-based global sensitivity analysis evaluates the relative importance of different input features affecting the model outputs for the DNN and LGB models under two scenarios (S1 and ST). The radar plots represent the normalized sensitivity indices, where the longer the radius, the more significant the feature’s contribution to the output’s variation. For both models, thermochemical factors such as ash content, temperature (T), volatile content (VC), and fixed carbon (FC) were mainly responsible for the changes, but their order of importance is different between the scenarios. The DNN has a more even sensitivity pattern, meaning there are more nonlinear interactions between features, whereas the LGB model displays more pronounced differences, implying a more substantial reliance on specific inputs. Additionally, hydrogen (H) and oxygen (O) have effects that depend on the scenario, indicating that the system behaves differently under various conditions.

**Fig. 8:**
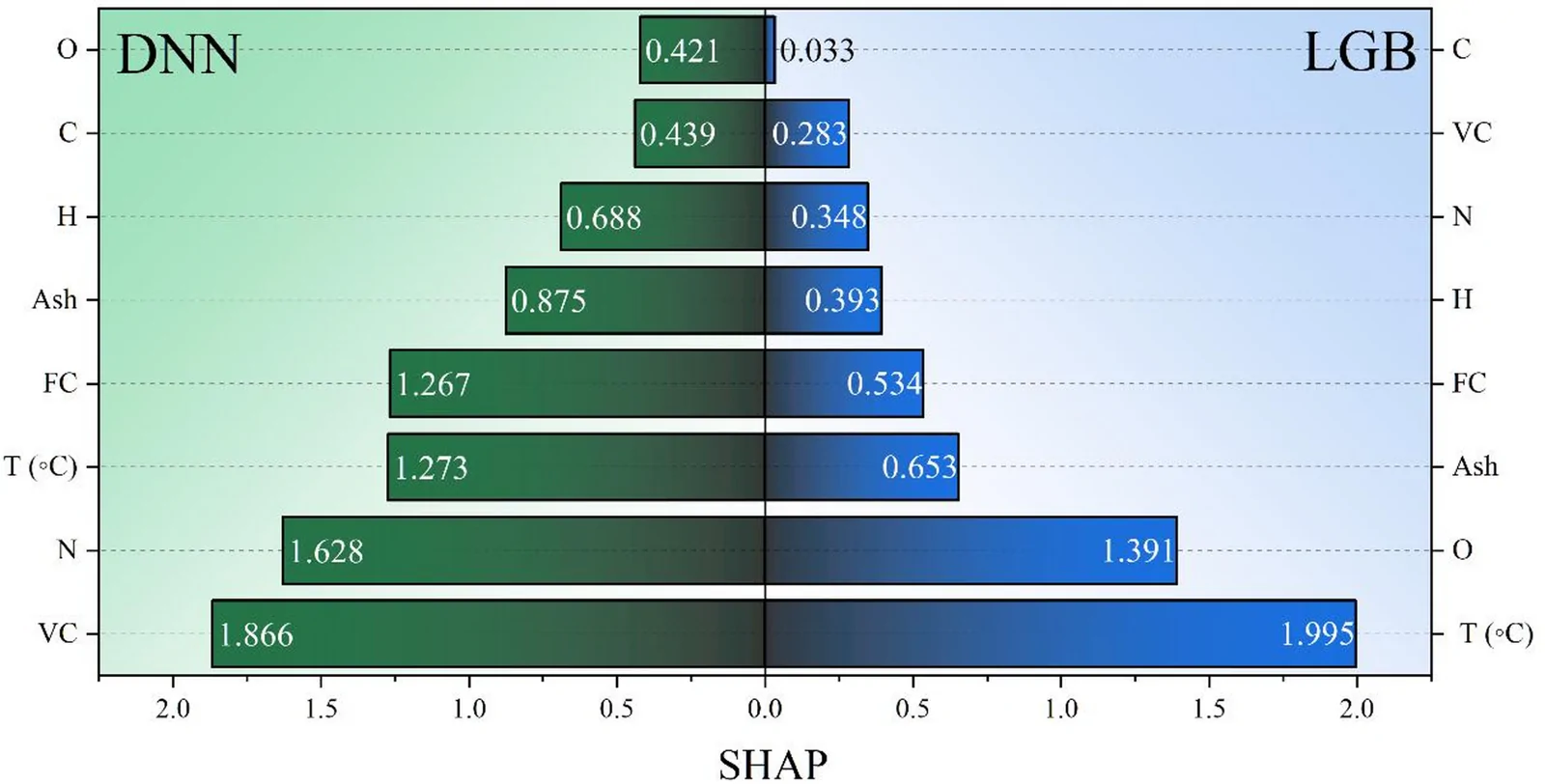
SHAP values highlighting the primary drivers of variation in the model output.

Figure 8 shows a comparative SHAP (Shapley Additive explanations) analysis of the DNN and LGB models, which displays how much each input feature contributed to explaining the changes in model predictions. The horizontal bar chart ranks the features according to their average absolute SHAP values, giving a clear, model-independent indication of feature importance. For both models, volatile content (VC) and operating temperature (T) are the two most important factors that determine predictive behavior. Still, a few differences in the magnitude and rank of the other features are noticed, revealing architectural variations between the DNN and LGB models. The DNN shows a more even spread of feature importance with contributions from FC, ash, and hydrogen, which might mean that the model has a better ability to understand complex nonlinear interactions. On the other hand, the LGB model shows more pronounced differences in importance scores, concentrating more strongly on the key variables.

The identified importance of elemental composition is consistent with the fundamental chemistry of biomass pyrolysis. Carbon content influences the carbonization process and the distribution of carbon between char and condensable products, thereby affecting bio-oil yield. Hydrogen content facilitates hydrogen-transfer reactions that stabilize free radicals generated during thermal decomposition, promoting the formation of condensable hydrocarbons and reducing excessive secondary cracking. In contrast, oxygen content governs the formation of oxygenated intermediates, including acids, aldehydes, ketones, and phenolic compounds, which originate during devolatilization and undergo subsequent secondary reactions. Higher oxygen contents generally increase the production of oxygenated volatiles while reducing the energy density of the resulting bio-oil. These elemental characteristics interact with pyrolysis temperature and volatile matter content, jointly determining the extent of primary devolatilization, vapor-phase cracking, and char formation, thereby explaining their influence on the predictive model and sensitivity rankings. This mechanistic interpretation provides physical support for the data-driven feature importance identified by the proposed framework.

**Fig. 9:**
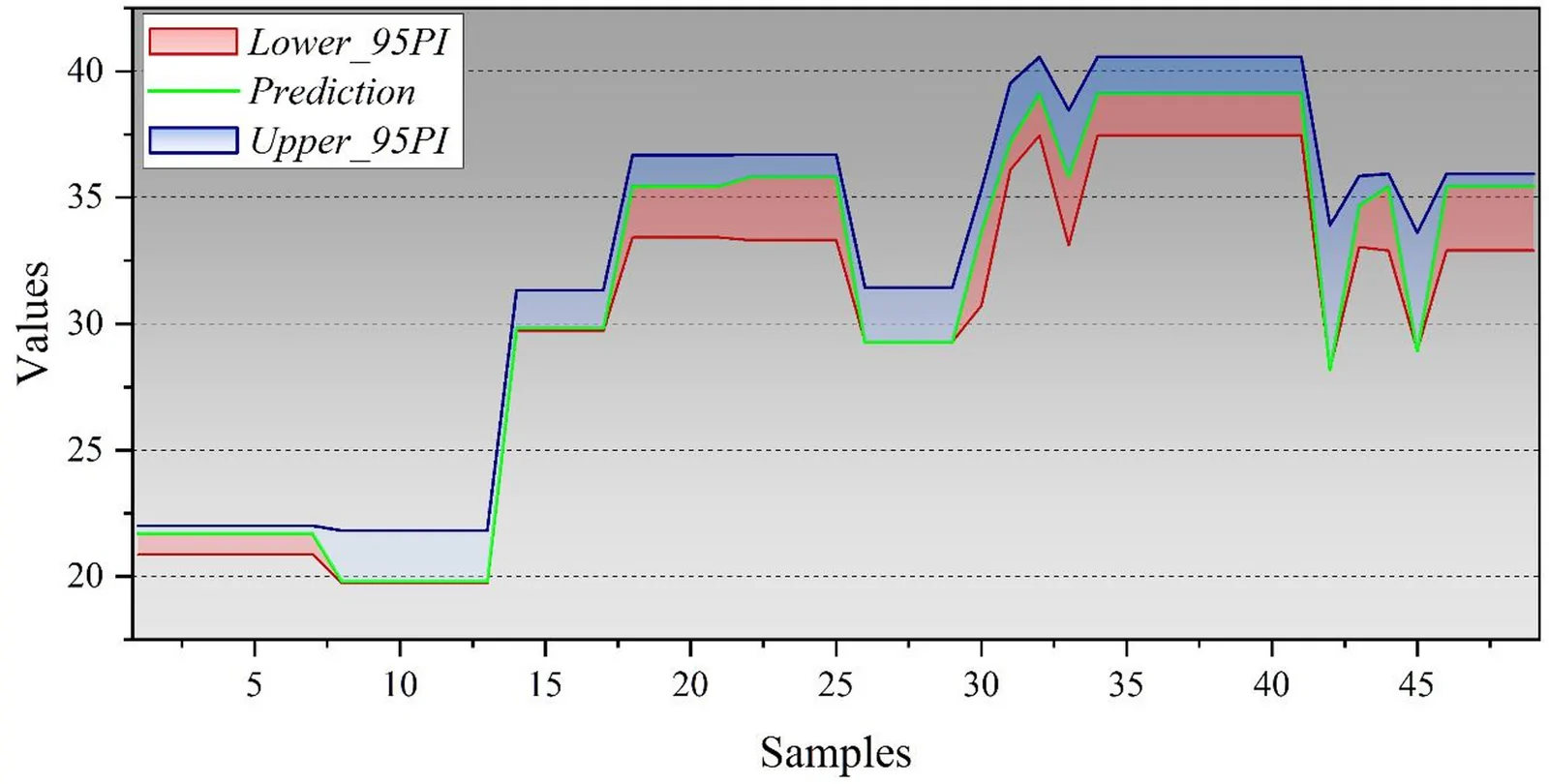
Prediction Interval Bounds (PIB) illustrating the uncertainty range surrounding model outputs, reflecting the confidence associated with the predicted values.

Figure 9 displays Prediction Interval Bounds (PIB), which represent the level of confidence with which the model’s predicted values are associated throughout the sequence of samples. The central line shows the point predictions, and the upper and lower 95% prediction interval limits identify the area where true observations should be if confidence is high. The short intervals in the quite stable areas signify that the predictions are highly trusted, while the rising bounds at times of sudden changes or the highest values indicate that the model is less certain due to the larger variability of the data. The predicted values are always kept within the 95% prediction intervals, thus are indicative of well-calibrated uncertainty and the correct behavior of the model. Also, the discontinuous changes and minor ripples in the figure provide evidence that the model can incorporate both slow changes and sudden shifts in the real process.

Table 4 presents a comparison of the run time among single, ensemble, and hybrid learning architectures, revealing the extra computational cost caused by the optimization-based extensions. The single models, DNN and LGBR, have the shortest run times, with LGBR showing better computational efficiency (0.03) than DNN (0.8). Nevertheless, the usage of metaheuristic optimization techniques considerably lengthens the run time for both models. In the case of DNN with Giant Armadillo Optimization and Mother Optimization Algorithm, the computational cost has been elevated to 825 and 870, respectively, reflecting the very high processing overheads produced. There are similar but less intense repercussions for LGBR hybrid models, where run time has been extended to 36 and 34, respectively. Even though there is an extra cost, the LGBR hybrid approaches are still drastically more efficient than the DNN ones.

**Table 4:** Execution time comparison among single, ensemble, and hybrid learning architectures.

Model variant	Time
DNN	0.8
DNN + Giant armadillo optimization	825
DNN + Mother optimization algorithm	870
LGBR	0.03
LGBR + Giant armadillo optimization	36
LGBR + Mother optimization algorithm	34

Table 5 presents the results of the single-sample non-parametric test that compares the performance distributions of the evaluated models and gives the statistical evidence of differences through the p-values and test statistics. For the baseline DNN and LGB models, the high p-values (0.3773 and 0.9141, respectively) imply that their performance distributions do not differ significantly from each other when the chosen non-parametric test is applied. However, the optimized DNN + GAO setup generates a p-value of 0.0213, indicating a statistically significant performance difference and implying that the Giant Armadillo Optimization drastically changes the model behavior. On the other hand, the DNN + MOA model variation does not present a distinct change in its performance, as can be seen in the high p-value of 0.2282. On the LGBR side, the GAO-based variant still looks statistically insignificant, while the LGBR + MOA variant shows a significant difference with a p-value equal to 0.0133.

**Table 5:** Singlewise non-parametric comparison of model performance distributions.

Difference of models	Parameter		Difference of models	Parameter	
	p_value	Statistic		p_value	Statistic
DNN	0.3773	13,510	LGB	0.9141	14,826
DNN + GAO	0.0213	12,298	LGB + GAO	0.7157	14,424
DNN + MOA	0.2282	13,163	LGBR + MOA	0.0133	12,108

## Practical implications

**Fig. 10:**
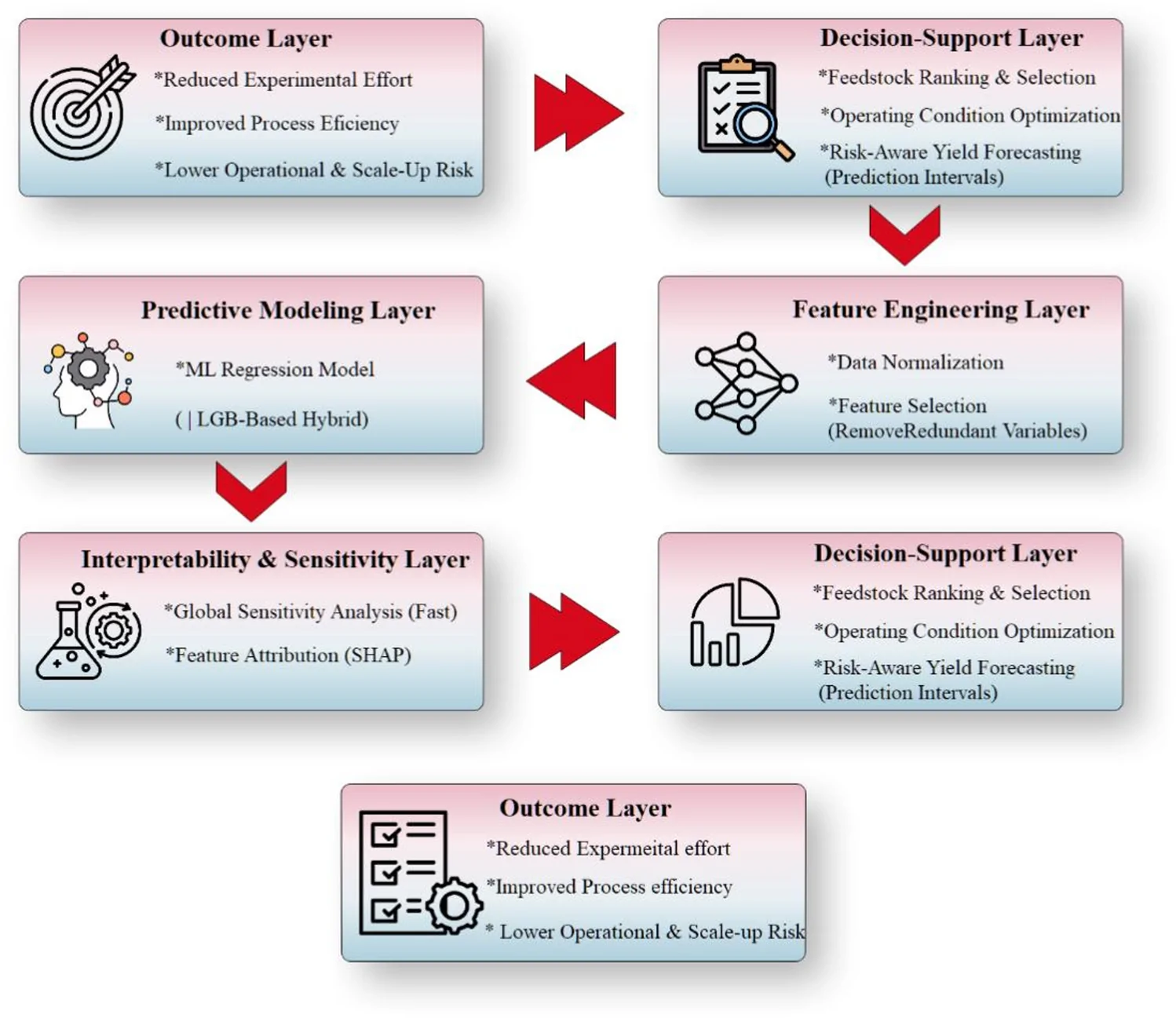
Workflow of the integrated ML framework from feature engineering and modeling to decision support and improved process outcomes.

The framework that combines feature-centric predictive and sensitivity analyses may produce several practical consequences, directly or indirectly, in industrial biomass pyrolysis, experimental design, and decision-making under uncertainty. Unlike solely accuracy-driven predictive models, this framework embeds feature selection, robust regression modeling, and global sensitivity analysis. As a result, it allows practitioners to predict bio-oil yield with a high level of confidence and to understand the rationale behind certain results. Both capabilities are highly valuable in the field of thermochemical conversion systems, where running cost, feedstock variation, and the risk of scaling up are the most important concerns. Overall flowchart of implications presented by Fig. 10.

### Application in feedstock selection and preprocessing

This framework’s first-hand practical application can be seen in decisions regarding feedstock screening and preprocessing. The volatile content (VC) and the elemental composition (especially carbon and oxygen) features were identified by the sensitivity and SHAP analyses as the major factors driving yield. Thus, operators will be able to rank their candidate biomass resources before performing expensive pilot-scale experiments. Those with higher VC and good elemental ratios will be given priority, and materials with too much ash or fixed carbon will be considered for blending or pretreatment.

Laboratories and industrial plants could use the trained model as a digital screening tool, where proximate and ultimate analysis data are input to quickly estimate the expected bio-oil yield together with its associated uncertainty. Such a practice allows them to decrease the reliance on trial-and-error experiments and to compress the time required for the development of the new biomass resource.

### Process optimization and operating condition control

This framework can be used as a tool for providing process optimization strategies in both direct and indirect ways, especially concerning the operating temperature. Global sensitivity indices and SHAP values reveal that temperature dominates the variability in bio-oil yield more than any other factor. Hence, process engineers can focus control efforts on temperature alone and keep it within the range where maximum bio-oil can be obtained, rather than distributing control actions across all parameters.

Besides that, the role of hydrogen and oxygen seems to vary with temperature and feedstock composition. Such trends in these two elements under different conditions may characterize the use of adaptive control strategies. Hence, the operating parameters can be changed dynamically based on the properties of the incoming batch instead of just using fixed points. The practical implementation of adaptive control will most likely be through the use of the supervisory control and data acquisition (SCADA) system or digital twins.

### Risk-aware decision-making and uncertainty management

The framework-derived prediction interval bounds (PIBs) serve as a quantitative level of prediction uncertainty, which is a crucial element in making risk-aware decisions. Narrow intervals point to stable operating regions, whereas wider intervals represent a higher level of uncertainty resulting from feature interactions or data sparsity. Plant operators and designers can use this information to identify the regimes where they can achieve the highest yields at the risk levels they are willing to accept.

When it comes to scale-up and techno-economic assessment, this modeling approach, with its uncertainty awareness, opens the door to scenario analysis. Thus, investors and third-party stakeholders can envisage best-case, nominal, and worst-case yield scenarios, which in turn serve as the basis for investment planning, sensitivity-based cost analysis, and contingency planning.

### Reduction of experimental and computational costs

Basically, the embedded feature selection step enables the model to lower the complexity by a great deal while maintaining excellent prediction performance (Table 6). Therefore, in real-world cases, less procurement and more reliable predictions will be realized, and, along with that, the analytical budget and the experimental data acquisition process become much more straightforward. Besides that, the top-notch performance-to-runtime ratio of the LightGB-based hybrid models implies that such a framework can be used for decision support almost in real time, even in scenarios where industrial facilities have limited resources.

**Table 6:** Practical mapping of framework outputs to real-world actions.

Framework component	Key output	Practical application	Expected benefit
Feature selection	Reduced, non-redundant feature set	Limits required laboratory analyses	Lower experimental cost and complexity
Predictive modeling (LGB/DNN)	High-accuracy yield prediction	Rapid yield estimation for new feedstocks	Faster decision-making
Global sensitivity analysis (FAST)	Ranking of influential variables	Identification of critical control parameters	Targeted process optimization
SHAP attribution	Directional feature impact	Understanding feature–yield relationships	Improved interpretability and trust
Prediction intervals	Quantified uncertainty bounds	Risk-aware operational planning	Reduced scale-up and operational risk

### Limitation of the study

One limitation of the present study is that the dataset was assembled from independent literature sources, where experimental metadata were not uniformly reported. Although biomass composition and pyrolysis operating conditions were consistently available, information such as reactor configuration, reactor scale, heating mechanism, feedstock geographic origin, and publication period was often incomplete or unavailable. These factors may introduce additional variability that the current predictive framework cannot explicitly capture. Future work would benefit from larger, standardized databases that include comprehensive experimental metadata, enabling the incorporation of reactor-specific variables into machine learning models. Such datasets would facilitate the development of models with improved transferability across different experimental setups and industrial-scale pyrolysis systems while further strengthening prediction reliability and generalizability.

## Conclusion

This study presented a thorough feature-based framework capable of predicting and interpreting the bio-oil yield from the pyrolysis of biomass. It equally emphasized the need for accurate predictions and transparent explanations for such complex thermochemical reactions. The findings suggested that the developed models can make highly successful predictions in the stages of training, validation, and testing; thus, the models are not only capable of handling familiar data but also new and unseen biomass samples and operating conditions. Moreover, the use of feature selection helped a great deal by limiting the duplication of information in arithmetic terms or input variables, thus helping the models to be more stable and the predicted yields to be more reliable. One of the significant contributions of this research was coupling a local sensitivity analysis with feature selection to quantitatively measure the effect of each process and feedstock variable on the output variable. In general, the sensitivity study showed that the most influential features for determining the bio-oil yield were the operating temperature and the volatile content of the biomass, highlighting the influence of these factors on the reaction mechanism and product composition during pyrolysis. It was also found that fixed carbon and ash content could affect the yield to some extent; thus, the effects of heat transfer and char production should be taken into account, and the roles of elemental hydrogen and oxygen were the least, occurring only under certain circumstances, reflecting the chemical nonlinearity of the system. Practically, the obtained results can be used by the biomass conversion industry to improve the processes at work in their production facilities. The well-defined main features that influence the final result allow operators to make necessary adjustments to the working parameters, select the raw materials that best suit the purpose, and plan the trials well, which can reduce the cost of the trials and the risk of operations. Besides that, an easy-to-understand framework also supports process monitoring, scaling up, and risk assessment when making decisions. In brief, this work connects data-driven prediction with physical understanding and thus provides a trustworthy decision-making tool for developing more efficient, sustainable, and economically viable bio-oil production systems.

## Supplementary Information

Below is the link to the electronic supplementary material.


Supplementary Material 1


## Data Availability

The data supporting the findings of this study are provided in the Supplementary Information accompanying this article.

## Abbreviations

DNN: Deep neural network
XAI: Explainable artificial intelligence
CNN: Convolutional neural network
LGBR: Light gradient boosting regression
SHAP: SHapley Additive exPlanations
H: Hydrogen
C: Carbon
GAO: Giant armadillo optimization
RMSE: Root mean square error
COV: Coefficient of variation
R²: Coefficient of determination
LGGA: LGBR + GAO
DNGA: DNN + GAO
GA: Genetic algorithm
PCC: Pearson correlation coefficient
XGBoost: Extreme gradient boosting
LIME: Local interpretable model-agnostic explanations
O: Oxygen
FC: Fixed carbon
VC: Volatile content
MOA: Mother optimization algorithm
MAPE: Mean absolute percentage error
MARD: Mean absolute relative difference
PIB: Prediction interval bounds
LGMO: LGBR + MOA
DNMO: DNN + MOA

## References

[1] Jabed, M. A. & Murad, M. A. A. Crop yield prediction in agriculture: A comprehensive review of machine learning and deep learning approaches, with insights for future research and sustainability. *Heliyon*. **10**, 24 (2024). 10.1016/j.heliyon.2024.e40836 PMC11667600 39720079

[2] Feng, W. et al. Covalent immobilization of phosphotungstic acid and amino acid on metal-organic frameworks with different structures: Acid–base bifunctional heterogeneous catalyst for the production of biodiesel from insect lipid. *Renew. Energy*. **210**, 26–39 (2023).

[3] Kang, Z. et al. Hydrogen transfer and reaction mechanism during in-situ pyrolysis of Fushun oil shale with steam injection. *Fuel* **381**, 133583 (2025).

[4] Ennaji, O., Hamma, A., Vergütz, L. & Allali, A. E. The assessment of soil variables relative importance for cereal yield prediction under rainfed cropping system in Morocco. *Smart Agric. Technol.* **11**, 100950 (2025).

[5] Sah, S., Haldar, D., Singh, R. N., Das, B. & Nain, A. S. Rice yield prediction through integration of biophysical parameters with SAR and optical remote sensing data using machine learning models. *Sci. Rep.* **14** (1), 21674 (2024). 39289440 10.1038/s41598-024-72624-4 PMC11408675

[6] Victor, B., Nibali, A. & He, Z. A systematic review of the use of deep learning in satellite imagery for agriculture. *IEEE J. Sel. Top. Appl. Earth Obs Remote Sens*, (2024).

[7] Li, C., Camac, J., Robinson, A. & Kompas, T. Predicting changes in agricultural yields under climate change scenarios and their implications for global food security. *Sci. Rep.* **15** (1), 2858 (2025). 39843615 10.1038/s41598-025-87047-y PMC11754462

[8] Wang, F. et al. Gas generation characteristics of synthetic ester insulating oils under thermal aging: influence of fatty acid chain structures. *IEEE Trans. Dielectr. Electr. Insul*. (2026).

[9] Zhang, Q. et al. Geochemical evolution and microbial controls on methanogenic potential in North China coalbed methane reservoirs: Implications for CBM bioengineering. *Fuel* **428**, 140384 (2027).

[10] Chatterjee, S., Mondal, S. K., Datta, A. & Gupta, H. K. Enhancing feature optimization for crop yield prediction models. *Curr. Agric. Res. J*. **12**, 2, (2024).

[11] Kick, D. R. et al. Yield prediction through integration of genetic, environment, and management data through deep learning, *G3 Genes Genomes Genet.* **13**(4), jkad006 (2023). 10.1093/g3journal/jkad006 PMC10085787 36625555

[12] Ashfaq, M., Khan, I., Shah, D., Ali, S. & Tahir, M. Predicting wheat yield using deep learning and multi-source environmental data. *Sci. Rep.* **15** (1), 26446 (2025). 40691684 10.1038/s41598-025-11780-7 PMC12280141

[13] Manzoor, M. F. A review of machine learning techniques for precision agriculture and crop yield prediction. *Prem J. Plant. Biol.* **1**, 100005 (2024).

[14] Miranda, M., Charfuelan, M., Toro, M. V. & Dengel, A. Informed learning for estimating drought stress at fine-scale resolution enables accurate yield prediction. *arXiv Prepr. arXiv2510.18648* (2025).

[15] Malashin, I. et al. Predicting sustainable crop yields: Deep learning and explainable AI tools. *Sustainability* **16** (21), 9437 (2024).

[16] Shawon, S. M., Ema, F. B., Mahi, A. K., Niha, F. L. & Zubair, H. T. Crop yield prediction using machine learning: An extensive and systematic literature review. *Smart Agric. Technol.* 100718 (2024).

[17] Li, H., Gao, J., Guo, Y. & Yuan, X. G. Application of XGBoost model and multi-source data for winter wheat yield prediction in Henan Province of China, *Big Data Inf. Anal.* **9**(bdia-09-002), 29–47 (2025).

[18] Najjar, H., Pathak, D., Nuske, M. & Dengel, A. Intrinsic explainability of multimodal learning for crop yield prediction. *arXiv Prepr. arXiv2508.06939* (2025).

[19] Chen, C. et al. Fast characterization of biomass pyrolysis oil via combination of ATR-FTIR and machine learning models. *Renew. Energy*. **194**, 220–231. 10.1016/j.renene.2022.05.097 (2022).

[20] Chen, C. et al. Combination of integrated machine learning model frameworks and infrared spectroscopy towards fast and interpretable characterization of model pyrolysis oil. *Renew. Energy*. **236**, 121434. 10.1016/j.renene.2024.121434 (2024).

[21] Omidkar, A., Alagumalai, A., Li, Z. & Song, H. Machine learning assisted techno-economic and life cycle assessment of organic solid waste upgrading under natural gas. *Appl. Energy*. **355**, 122321 (2024).

[22] Hinton, G. E., Osindero, S. & Teh, Y. W. A fast learning algorithm for deep belief nets. *Neural Comput.* **18** (7), 1527–1554 (2006). 16764513 10.1162/neco.2006.18.7.1527

[23] Alzamzami, F., Hoda, M. & Saddik, A. E. Light gradient boosting machine for general sentiment classification on short texts: a comparative evaluation. *IEEE Access.* **8**, 101840–101858 (2020).

[24] Kyrou, G., Charilogis, V. & Tsoulos, I. G. Improving the giant-armadillo optimization method. *Analytics* **3** (2), 225–240 (2024).

[25] Alsayyed, O. et al. Giant armadillo optimization: A new bio-inspired metaheuristic algorithm for solving optimization problems. *Biomimetics* **8** (8), 619 (2023). 38132558 10.3390/biomimetics8080619 PMC10741582

[26] Santana, D. et al. Interactions of four teiid lizards with giant armadillo burrows and range extension for two endemic cerrado species. *Rev Latinoam. Herpetol*, **6**(3), e709–e121.

[27] Matoušová, I., Trojovský, P., Dehghani, M., Trojovská, E. & Kostra, J. Mother optimization algorithm: A new human-based metaheuristic approach for solving engineering optimization. *Sci. Rep.* **13** (1), 10312 (2023). 37365283 10.1038/s41598-023-37537-8 PMC10293246

[28] Chicco, D., Warrens, M. J. & Jurman, G. The coefficient of determination R-squared is more informative than SMAPE, MAE, MAPE, MSE and RMSE in regression analysis evaluation. *PeerJ Comput. Sci.* **7**, e623 (2021). 34307865 10.7717/peerj-cs.623 PMC8279135

[29] Montgomery, D. C., Peck, E. A. & Vining, G. G. *Introduction to Linear Regression Analysis* (Wiley, 2021).

[30] Hyndman, R. J. & Koehler, A. B. Another look at measures of forecast accuracy. *Int. J. Forecast.* **22** (4), 679–688 (2006).

[31] Everitt, B. S. & Skrondal, A. *The Cambridge Dictionary of Statistics*, vol. 4 (Cambridge University Press, 2010).

[32] Freckmann, G. et al. Mean absolute relative difference of blood glucose monitoring systems and relationship to ISO 15197. *J. Diabetes Sci. Technol.* **16** (5), 1089–1095 (2022). 33759584 10.1177/19322968211001402 PMC9445334

